# Higher-order topological Mott insulator on the pyrochlore lattice

**DOI:** 10.1038/s41598-021-99213-z

**Published:** 2021-10-12

**Authors:** Yuichi Otsuka, Tsuneya Yoshida, Koji Kudo, Seiji Yunoki, Yasuhiro Hatsugai

**Affiliations:** 1 Computational Materials Science Research Team, RIKEN Center for Computational Science (R-CCS), Kobe, Hyogo 650-0047 Japan; 2 Quantum Computational Science Research Team, RIKEN Center for Quantum Computing (RQC), Wako, Saitama 351-0198 Japan; 3 Graduate School of Pure and Applied Sciences, University of Tsukuba, Tsukuba, Ibaraki 305-8571 Japan; 4 Department of Physics, University of Tsukuba, Tsukuba, Ibaraki 305-8571 Japan; 5 Computational Condensed Matter Physics Laboratory, RIKEN, Wako, Saitama 351-0198 Japan; 6 Computational Quantum Matter Research Team, RIKEN Center for Emergent Matter Science (CEMS), Wako, Saitama 351-0198 Japan

**Keywords:** Condensed-matter physics, Quantum physics

## Abstract

We provide the first unbiased evidence for a higher-order topological Mott insulator in three dimensions by numerically exact quantum Monte Carlo simulations. This insulating phase is adiabatically connected to a third-order topological insulator in the noninteracting limit, which features gapless modes around the corners of the pyrochlore lattice and is characterized by a ${\mathbb {Z}}_{4}$ spin-Berry phase. The difference between the correlated and non-correlated topological phases is that in the former phase the gapless corner modes emerge only in spin excitations being Mott-like. We also show that the topological phase transition from the third-order topological Mott insulator to the usual Mott insulator occurs when the bulk spin gap solely closes.

## Introduction

Nontrivial topological properties and many-body effects are the two major subjects in modern condensed matter physics. In a system involving these two subjects, obtaining knowledge of a wave function, often required for characterizing topological properties, is difficult and demanding because of the many-body nature. In such a situation, the adiabatic-connection approach and the notion of bulk-edge correspondence can still provide smoking-gun evidence for an interacting topological phase.

The topological Mott insulator (TMI) is a novel state of matter in which nontrivial topological properties and correlation effects coexist^1,2^ (Note that the topological Mott insulators studied here and in Ref.^3^ are different, although the same term is used. In the former case, the topological Mott insulator is found as a Mott insulator possessing gapless edge spin-only excitations, while in the latter case band topology is induced from spontaneous symmetry breaking due to interactions.). Such a state was first proposed by Pesin and Balents as one of possible ground states for Ir-based pyrochlore oxides^4^. Among various interesting issues originated in their proposal, gapless surface spin-only excitations in the TMI are intriguing, since it is in sharp contrast to the case of the usual topological insulators where gapless edge excitations appear in the single-particle spectrum^5–7^. Namely, in the TMI, the bulk-edge (boundary) correspondence^8,9^, one of the most distinguished and ubiquitous properties of the topological insulators, is generalized by the correlation effect. Soon after the proposal, intensive studies have examined the possibility of TMI in several condensed matter systems^10–20^, yielding concrete evidences for one^17^ and two^18–20^ dimensional cases. However, the TMI in three dimensions (3D) has not yet been fully explored, partly because of lack of reliable methods to study the correlated systems in 3D such as the complicated model considered for the Ir oxides^4^.

On the one hand, recently, another type of unconventional topological insulators, a higher-order topological insulator (HOTI), has been attracting increasing interest^21,22^. The *n*th-order topological insulator in *d*-dimensions features gapless excitations around its ($d-n$)-dimensional boundaries. Thus, also in HOTI, the bulk-edge correspondence is generalized. The studies of the HOTI have not always been material-oriented^23–28^, but also have covered a wide range of models^21,22,29–49^ and experimental setups^50–57^. Among them, three of the present authors proposed a tailored model to investigate the correlation effects on the HOTI in $d=2$ and found a correlated topological state dubbed as a higher-order topological Mott insulator (HOTMI), in which gapless corner modes emerge only in spin excitations^45^.

In this study, we present unbiased numerical evidence for a HOTMI in 3D by constructing a repulsive Hubbard model with spin-dependent hoppings on the pyrochlore lattice. Our results support the TMI in 3D in the sense that, both in the TMI and the HOTMI, the nontrivial bulk topological property manifests itself in the edge states only through the spin channel. As in the case of the kagome lattice^45^, the repulsive Hubbard model with spin-dependent hoppings on the pyrochlore lattice can be mapped into the attractive Hubbard model by the particle-hole transformation, and hence we can utilize a quantum Monte Carlo (QMC) method for the correlated model in 3D without facing the negative-sign problem. We show that the on-site interaction (*U*) added to the HOTI closes neither the charge nor spin gap in the bulk, which suggests that the higher-order topology characterized by a ${\mathbb {Z}}_{4}$ spin-Berry phase^58^ in the HOTI is adiabatically preserved in the $U>0$ phase. As for the properties around the boundaries, the characteristic gapless corner modes are found only in the spin sector. These results indicate that the $U>0$ phase, next to the HOTI, is the third-order topological Mott insulator in 3D. We also show that the gapless corner modes disappear when the bulk spin gap vanishes at a phase boundary between the HOTMI and the Mott insulator (MI).

## Results

### Model

**Figure 1:**
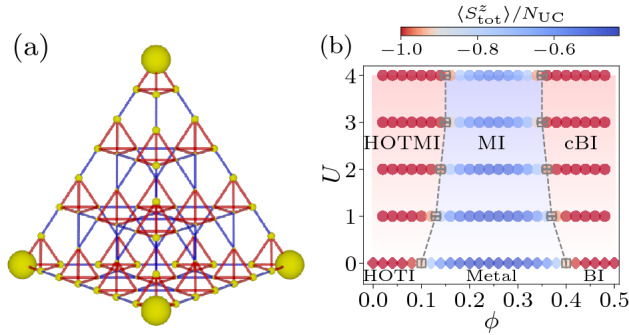
(**a**) Pyrochlore lattice for $L=4$ with the open boundary conditions. The small upward tetrahedron represents the unit cell. Transfer integral for the intra (inter) unit cell indicated by red (blue) is $t_{\bigtriangleup }$ ($t_{\bigtriangledown }$). Enhancement of the local moment by the on-site interaction $U=1$, i.e., $\langle m_{i}^{2} \rangle _{U=1} - \langle m_{i}^{2} \rangle _{U=0}$, is shown by the radius of the yellow spheres for $\phi =0.08$ and $T=0.08$. (**b**) Ground-state phase diagram as function of $\phi$ and *U*. The color of the two symbols, circles for $U>0$ and diamonds at $U=0$, represents the value of $\langle S_{\text {tot}}^{z} \rangle /N_{\text {UC}}$.

We study the spinful interacting model on the pyrochlore lattice. The Hamiltonian is described by

1 $$\begin{aligned} {\mathcal {H}} = {\mathcal {H}}_{t}^{\bigtriangleup } + {\mathcal {H}}_{t}^{\bigtriangledown } + {\mathcal {H}}_{U} - \mu \, N - h \, S_{\text {tot}}^{z}, \end{aligned}$$

with

2 $$\begin{aligned} {\mathcal {H}}_{t}^{\Gamma } = - t_{\Gamma } \sum _{i,j \in \Gamma } \sum _{\alpha , \beta = \uparrow , \downarrow } \left( c_{i \alpha }^{\dagger } \sigma _{\alpha \beta }^{z} c_{j \beta } + \text {h.c.} \right), \end{aligned}$$

and

3 $$\begin{aligned} {\mathcal {H}}_{U} = U \sum _{i} \left( n_{i \uparrow } - \frac{1}{2} \right) \left( n_{i \downarrow } - \frac{1}{2} \right), \end{aligned}$$

where $c_{i \alpha }^{\dagger }$ creates an electron with spin $\alpha$ $(=\uparrow , \downarrow )$ at site *i*, $\sigma _{\alpha \beta }^{z}$ is the *z*-component of Pauli matrix, and $n_{i \alpha }=c_{i \alpha }^{\dagger }c_{i \alpha }$ is a number operator. Since we consider the model in the grand canonical ensemble, we explicitly include the terms for a chemical potential $\mu$ and a magnetic field *h*, which are coupled to the total number of the electrons, $N=\sum _{i \alpha } n_{i \alpha }$, and the total magnetization, $S_{\text {tot}}^{z}=\sum _{i}\left( n_{i \uparrow } - n_{i \downarrow }\right) /2$. In the kinetic part ${\mathcal {H}}_{t}^{\Gamma }$ with $\Gamma =\bigtriangleup$ and $\bigtriangledown$, $t_{\bigtriangleup }$ and $t_{\bigtriangledown }$ denotes the transfer integrals for the intra and inter unit cell, respectively (see Fig. 1a). Their relative ratio is parameterized by $\phi$ with $0\le \phi \le 1/2$ as $t_{\bigtriangleup } = t \sin \left( \phi \, \pi \right)$ and $t_{\bigtriangledown } = t \cos \left( \phi \, \pi \right)$. Here, *t* is chosen as an energy unit, namely, $t=1$. The Hubbard term of Eq. (3) represents the repulsive ($>0$) on-site interaction.

The system preserves a certain type of particle-hole symmetry defined by a transformation of $c_{i \uparrow } \rightarrow {\tilde{c}}_{i \downarrow }^{\dagger }$ and $c_{i \downarrow } \rightarrow {\tilde{c}}_{i \uparrow }^{\dagger }$, under which the Hamiltonian is invariant for $\mu =0$. The number of electrons with spin up, $\langle n_{i \uparrow } \rangle$, where $\langle \cdots \rangle$ denotes an expectation value defined below, is related to that for spin down in the transformed Hamiltonian as $\langle n_{i \uparrow } \rangle = 1 - \langle {\tilde{n}}_{i \downarrow } \rangle$. Since the invariant Hamiltonian trivially yields the same expectation value, $\langle {\tilde{n}}_{i \downarrow } \rangle = \langle n_{i \downarrow } \rangle$, the system is half filled, i.e., $\langle n_{i \uparrow } \rangle + \langle n_{i \downarrow } \rangle =1$, at $\mu =0$, which is the case we consider in this study.

The unusual ingredient in our model would be $\sigma _{\alpha \beta }^{z}$ in Eq. (2), which simply leads to the spin-dependent transfer integrals. Such a modification of the model was proposed in the previous study^45^ for the kagome lattice to induce the bulk gap in the single-particle spectrum, necessary for realizing the topological phases. In this study, $\sigma _{\alpha \beta }^{z}$ is also crucial for allowing the sign-problem-free QMC calculations.

We employ the finite-temperature auxiliary-field quantum Monte Carlo method^59–63^. An expectation value of a physical operator ${\mathcal {O}}$ at a finite temperature *T* is calculated in the grand canonical ensemble as $\langle {\mathcal {O}} \rangle = \frac{1}{Z} \text {Tr} \left( {\mathcal {O}} \ e^{-\beta {\mathcal {H}}} \right)$, where $Z=\text {Tr}\left( e^{-\beta {\mathcal {H}}}\right)$ is the partition function, and $\beta =1/T$ denotes an inverse temperature. To be convinced that our model is sign-problem free, let us consider a partial particle-hole transformation, $c_{i \uparrow } \rightarrow {\tilde{c}}_{i \uparrow }$ and $c_{i \downarrow } \rightarrow {\tilde{c}}_{i \downarrow }^{\dagger }$, which maps the Hamiltonian into the following form (excluding a constant term):

4 $$\begin{aligned} {\mathcal {H}} \rightarrow \tilde{{\mathcal {H}}} =&- \sum _{\Gamma = \bigtriangleup , \bigtriangledown } \sum _{i,j \in \Gamma } \sum _{\alpha = \uparrow , \downarrow } t_{\Gamma } \left( {\tilde{c}}_{i \alpha }^{\dagger } {\tilde{c}}_{j \alpha } + \text {h.c.} \right) \nonumber \\&- U \sum _{i} \left( {\tilde{n}}_{i \uparrow } - \frac{1}{2} \right) \left( {\tilde{n}}_{i \downarrow } - \frac{1}{2} \right) \nonumber \\&- \mu \sum _{i} \left( {\tilde{n}}_{i \uparrow } - {\tilde{n}}_{i \downarrow } \right) - \frac{h}{2} \sum _{i} \left( {\tilde{n}}_{i \uparrow } + {\tilde{n}}_{i \downarrow } \right). \end{aligned}$$

This reads the attractive Hubbard model without the spin-dependency in the transfer integrals, therefore being free from the sign problem in the absence of the effective magnetic field, namely $\mu =0$^64^. It is also understood that $\langle S_{\text {tot}}^{z} \rangle$ is nonzero even for $h=0$, because in terms of the attractive model, the zero chemical potential does not correspond to the half filling for non-bipartite lattices^65^. Owing to the absence of the negative sign problem, we can perform the QMC simulations for fairly large clusters with several hundreds of the lattice sites far beyond the scope of the exact diagonalization method. To study the bulk and boundary properties, we treat the model under periodic boundary conditions (PBC) and open boundary conditions (OBC). The total number of the unit cells $N_{\text {UC}}$ is $L^{3}$ for PBC and $L(L+1)(L+2)/6$ for OBC, where *L* denotes the number of the unit cells aligned in the linear dimension (see Fig. 1a for the case of OBC), and the total number of the lattice sites $N_{\text {s}}$ is $4N_{\text {UC}}$.

### Phase diagram

The model for $U>0$ has three different phases; the HOTMI, the MI, and the correlated band insulator (cBI) as summarized in Fig. 1b. Here, the cBI is the trivial band insulator with the charge and spin gaps, thus being different from the HOTMI or the MI. The two phase boundaries, referred to as $\phi _{\text {c}1}^{U}$ and $\phi _{\text {c}2}^{U}$, are determined as points where the value of $\langle S_{\text {tot}}^{z} \rangle / N_{\text {UC}}$ deviates from $-1$, which is the value of that in the HOTI or the band insulator (BI) at $U=0$ (see Supplemental Material). This is because the HOTMI (cBI) is smoothly connected from the HOTI (BI) and is therefore labeled by the same value of $\langle S_{\text {tot}}^{z} \rangle$. The phase boundaries thus determined are legitimated by calculating a more direct quantity, i.e., the spin gap, from magnetization plateaus under the nonzero magnetic field *h* (see Supplemental Material).

### HOTI at $U=0$

There are three phases at $U=0$ when $\phi$ is varied: the HOTI, the metal, and the BI, divided by $\phi _{\text {c}1}^{0} \simeq 0.1$ and $\phi _{\text {c}2}^{0} \simeq 0.4$. In the limit of $\phi =0$ or 1/2, the system is completely decoupled into a set of isolated tetrahedrons, where the energy levels in each tetrahedron is $E=-3$ (3) and 1 (− 1) for up (down) spin with the latter being threefold degenerate. Consequently, both of the HOTI and the BI have $\langle S_{\text {tot}}^{z} \rangle /N_{\text {UC}}= -1$, since the chemical potential is set as $\mu =0$. The difference between the two gapped phases can be determined by the ${\mathbb {Z}}_4$ spin-Berry phase^58^: $\gamma =\pi$ for the HOTI and $\gamma =0$ for the BI. This topological invariant is defined by an integration of the many-body Berry connection associated with local gauge twists. The $S_{4}$ symmetry of the pyrochlore lattice yields its ${\mathbb {Z}}_4$ quantization as $\gamma =2\pi n/4$ with $n=0,1,2,3$ (see Supplemental Material).

The distinction between the HOTI and the BI can also be made by imposing the OBC, since according to the bulk-edge correspondence^8,9^ the topological property in the bulk is reflected in the edge states. The edge states of the HOTI appear as the zero-energy states in the energy spectra, whereas such states are absent in the BI^33^ (see Supplemental Material). The zero-energy states are fourfold degenerate for each spin, originating from the isolated sites at the four corners of the finite-size cluster of the pyrochlore lattice (see Fig. 1a) in the limit of $\phi =0$. Therefore, the zero-energy states for $\phi < \phi _{\text {c}1}^{0}$ are mostly localized at these corners (see Supplemental Material), representing the third-order topological insulator in 3D^33^.

### From HOTI to HOTMI

**Figure 2:**
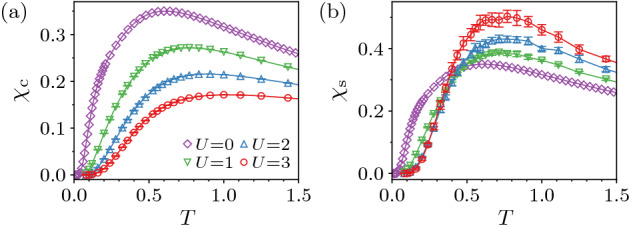
Temperature dependence of (**a**) charge compressibility $\chi _{\text {c}}$ and (**b**) spin susceptibility $\chi _{\text {s}}$ at $\phi =0.08$ for $L=5$ under the PBC.

**Figure 3:**
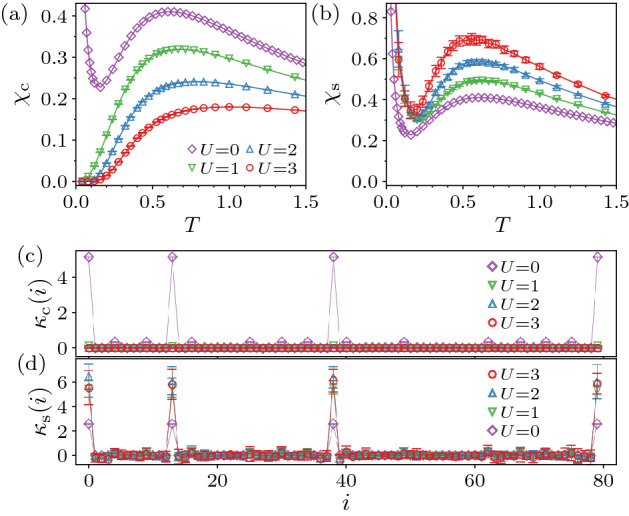
Temperature dependence of (**a**) charge compressibility $\chi _{\text {c}}$ and (**b**) spin susceptibility $\chi _{\text {s}}$ at $\phi =0.08$ for $L=5$ under the OBC. Site-resolved (**c**) charge compressibility $\kappa _{\text {c}}(i)$ and (**d**) spin susceptibility $\kappa _{\text {s}}(i)$ for the system of $L=4$ under the OBC at $\phi =0.08$ and $T=0.08$.

The HOTI changes to the HOTMI when the on-site interaction *U* is turned on. It is, however, difficult to distinguish these two phases by the bulk properties because they both have the charge and spin gaps. In Fig. 2, we show temperature dependence of the charge compressibility $\chi _{\text {c}}$ and the spin susceptibility $\chi _{\text {s}}$, defined respectively as

5 $$\begin{aligned} \chi _{\text {c}} = \frac{1}{N_{\text {s}}} \frac{\partial \langle N \rangle }{\partial \mu }, \end{aligned}$$

and

6 $$\begin{aligned} \chi _{\text {s}} = \frac{1}{N_{\text {s}}} \frac{\partial \langle S_{\text {tot}}^{z} \rangle }{\partial h}, \end{aligned}$$

at $\phi =0.08<\phi _{\text {c}1}^{0}$. Except that $\chi _{\text {c}}$ is more strongly suppressed by *U*, there is no obvious qualitative difference between the HOTI and the HOTMI. On the other hand, if we consider the system under the OBC, the difference can be noticeable as shown in Fig. 3. At $U=0$, both $\chi _{\text {c}}$ and $\chi _{\text {s}}$ show a diverging behavior at low *T*, which is due to the gapless modes in the HOTI. For $U>0$, the gapless charge excitations vanish as shown in Fig. 3a, whereas the gapless spin excitations remain as evident in the diverging behavior of $\chi _{\text {s}}$ for $U>0$ (see Fig. 3b). The feature that the boundary states posses only the charge gap seems common in the TMI^1,2^.

The gapless modes observed from $\chi _{\text {c}}$ and $\chi _{\text {s}}$ for the system under the OBC are elucidated by “site-resolved” charge compressibility and spin susceptibility, defined respectively as

7 $$\begin{aligned} \kappa _{\text {c}}(i) = \frac{\partial \langle n_{i} \rangle }{\partial \mu }, \end{aligned}$$

and

8 $$\begin{aligned} \kappa _{\text {s}}(i) = \frac{\partial \langle m_{i} \rangle }{\partial h}, \end{aligned}$$

with $m_{i} = \left( n_{i \uparrow } - n_{i \downarrow } \right) /2$, which are similar to a momentum-resolved compressibility^66–68^. As shown in Fig. 3c, $\kappa _{\text {c}}(i)$ for $U=0$ exhibits peaks at four site locations that are the isolated corners in the limit of $\phi = 0$. This is the expected behavior of the third-order topological insulator in three dimensions. Note that the peaks in $\kappa _{\text {s}}(i)$ of Fig. 3d are identical to those in $\kappa _{\text {c}}(i)$ (except for the constant factor) at $U=0$ because the gapless excitations appears in the single-particle spectrum. The peaks in $\kappa _{\text {c}}(i)$ immediately disappear upon inclusion of *U*, while the peaks in $\kappa _{\text {s}}(i)$ remain and even develop for $U>0$. This clearly shows that the gapless spin excitations appear around the ($d-3$)-dimensional boundary, namely the corners, which can also be observed from the enhancement of the local magnetic moments $\langle m_{i}^{2} \rangle - \langle m_{i}^{2} \rangle _{0}$, where $\langle \cdots \rangle _{0}$ denotes the expectation value for $U=0$, as shown in Fig. 1a.

It is desired to calculate some quantity which directly characterizes the topological index such as the spin-Berry phase^58^ for further identifying the $U>0$ phase as the HOTMI. However, such calculation is not feasible because there is no established way within the framework of the auxiliary-field QMC. It is also because the system size of the pyrochlore lattice is too large to apply the exact diagonalization method, which was possible for the kagome lattice^45^. Nevertheless, it is reasonable to consider that the nontrivial topology is protected by the bulk charge and spin gaps as shown in Fig. 2.

### Collapse of the HOTMI

**Figure 4:**
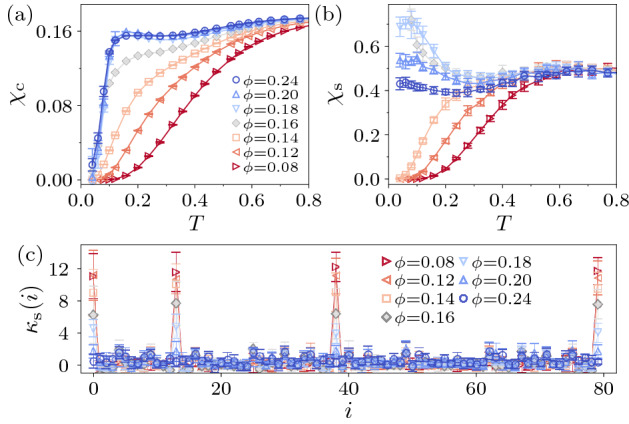
Temperature dependence of (**a**) charge compressibility $\chi _{\text {c}}$ and (**b**) spin susceptibility $\chi _{\text {s}}$ for the system of $L=5$ under the PBC at $U=3$. (**c**) Site-resolved spin susceptibility $\kappa _{\text {s}}(i)$ for $L=4$ under the OBC at $U=3$ and $T=0.08$. The critical point estimated by the value of $\langle S_{\text {tot}}^{z} \rangle /N_{\text {UC}}$ is $\phi _{\text {c}1}^{U=3} \simeq 0.16$.

Next, we examine how the HOTMI evolves into the MI with varying $\phi$ at a fixed value of $U=3$. We confirm in Fig. 4a that the charge gap does not close between the HOTMI and the MI, since the temperature dependence of $\chi _{\text {c}}$ always shows the thermally-activated behavior below and above $\phi _{\text {c}1}^{U} \simeq 0.16$ that is determined by $\langle S_{\text {tot}}^{z} \rangle /N_{\text {UC}}$. In addition, the change of $\chi _{\text {c}}$ with increasing $\phi$ is found to be nonuniform. Below $\phi _{\text {c}1}^{U}$, $\chi _{\text {c}}$ gradually increases as $\phi \rightarrow \phi _{\text {c}1}^{U}$, indicating that the charge gap continuously decreases. At $\phi =\phi _{\text {c}1}^{U}$, the temperature dependence of $\chi _{\text {c}}$ qualitatively changes, and they fall into the almost same curve for $\phi >\phi _{\text {c}1}^{U}$, which suggests that the charge gap in the MI does not depend on $\phi$. This abrupt change in $\chi _{\text {c}}$ implies that the natures of the charge gaps are different between the HOTMI and the MI. In Fig. 4b, it is observed that the thermally-activated behavior of $\chi _{\text {s}}$ is completely lost for $\phi >\phi _{\text {c}1}^{U}$. The peak structure in $\kappa _{\text {s}}(i)$ also vanishes when the spin gap closes at $\phi _{\text {c}1}^{U}$ as shown in Fig. 4c (see Supplemental Material). This topological phase transition is intrinsically different form the noninteracting counterpart; while in the noninteracting systems the topological property can change when the charge and spin gaps close, here the topological phase transition occurs when the spin gap solely closes.

## Discussion

Finally, we comment on possible realizations of the HOTMI. The starting model of Eq. (1) involves the spin-dependent transfer integrals which seems difficult to realize in material. However, if we exploit the mapping of Eq. (4), the mapped attractive model turns out to have the hoppings which does not dependent on the spin. We thus expect that the HOTMI would be realized in materials with the breathing pyrochlore lattice structure and the attractive interaction at quarter filling. Such a system may also have instability to superconductivity (The superconducting phase in terms of the attractive model corresponds to a ferromagnetic state in the $xy$ plane, which should emerge somewhere in the MI region of the phase diagram.). Then, we are also tempted to speculate that some aspects of the HOTMI on the kagome lattice^45^ might be related to recently discovered kagome superconductors $\hbox {AV}_{3}\hbox {Sb}_{5}$ (A = K, Rb, Cs)^69–72^.

We have studied the spinful Hubbard-like model on the pyrochlore lattice in three dimensions. Owing to the well-designed amendment of the model, namely the spin-dependent transfer integrals originally proposed in the previous study on the kagome lattice, the model yields the higher-order topological insulator in the noninteracting limit. The spin-dependent transfer integrals also enable us to study the model by the auxiliary-field quantum Monte Carlo method, which is numerically exact, without suffering the negative-sign problem. With including the interaction *U*, we have found that the gapless corner spin-only excitations persist for the system with the open boundaries, while the bulk hosts both the charge and spin gaps, which is characteristics of the topological Mott insulator. To our best knowledge, this is the first unbiased evidence for the topological Mott insulator in three dimensions. Furthermore, we have confirmed that this phase also falls within the category of the higher-order topological Mott insulator by calculating the site-resolved spin susceptibility showing the peaks at the corners. The higher-order topological Mott insulator collapses into the usual Mott insulator when the bulk spin gap solely closes.

## Supplementary Information

Supplementary Information.

## Data Availability

The datasets generated and/or analyzed during the current study are available from the corresponding author on reasonable request.
