# Supplementary material for: Higher-order topological Mott insulator on the pyrochlore lattice
Source: Sci Rep. 2021 Oct 12;11:20270. doi: 10.1038/s41598-021-99213-z (PMC8511174; doi:10.1038/s41598-021-99213-z)
Supplement: Supplementary file 1 — Supplementary Information. [file 41598_2021_99213_MOESM1_ESM.pdf]

Supplemental Material: Higher-Order Topological Mott Insulator on the Pyrochlore Lattice

Yuichi Otsuka,[1,2] Tsuneya Yoshida,[3,4] Koji Kudo,[4] Seiji Yunoki,[1,2,5,6] and Yasuhiro Hatsugai[3,4]

[1] *Computational Materials Science Research Team, RIKEN Center for Computational Science (R-CCS), Kobe, Hyogo 650-0047, Japan*
[2] *Quantum Computational Science Research Team, RIKEN Center for Quantum Computing (RQC), Wako, Saitama 351-0198, Japan*
[3] *Graduate School of Pure and Applied Sciences, University of Tsukuba, Tsukuba, Ibaraki 305-8571, Japan*
[4] *Department of Physics, University of Tsukuba, Tsukuba, Ibaraki 305-8571, Japan*
[5] *Computational Condensed Matter Physics Laboratory, RIKEN, Wako, Saitama 351-0198, Japan*
[6] *Computational Quantum Matter Research Team, RIKEN Center for Emergent Matter Science (CEMS), Wako, Saitama 351-0198, Japan*

S1. $\mathbb{Z}_4$ SPIN-BERRY PHASE

In this appendix, we describe the definition of the spin-Berry phase γ and discuss its $\mathbb{Z}_4$ quantization [S1, S2]. The spin-Berry phase is given by an integration over the angles of local gauge twist defined below. Let us consider the bulk system on the pyrochlore lattice. Picking up a specific downward tetrahedron, we define a unitary operator as

$$U_-(\vec{\theta}) = \exp\left\{ i \sum_{j=1}^{4} n_j^- \phi_j \right\}, \tag{S1}$$

where $j = 1, \cdots, 4$ is the site index of the tetrahedron, $n_j^- = n_{j\uparrow} - n_{j\downarrow}$, $\phi_j = \sum_{k=1}^{j} \theta_k$, and $\vec{\theta} = (\theta_1, \theta_2, \theta_3, \theta_4)$ is a four-dimensional parameter defined on a torus T^4. Setting $\mu = h = 0$ in Eq. (1), we then rewrite the Hamiltonian as

$$\mathcal{H}(\vec{\theta}) = \mathcal{H}_t^{\triangle} + U_-(\vec{\theta})\mathcal{H}_t^{\nabla} U_-^{\dagger}(\vec{\theta}) + \mathcal{H}_U. \tag{S2}$$

This modification brings the Peierls phase in the hopping term within the chosen downward tetrahedron as shown in Fig. S1(a). Let us define the spin-Berry connection as

$$\vec{A}(\vec{\theta}) = \langle G(\vec{\theta}) | \vec{\nabla}_{\vec{\theta}} | G(\vec{\theta}) \rangle, \tag{S3}$$

where $\vec{\nabla}_{\vec{\theta}} = (\partial/\partial\theta_1, \cdots, \partial/\partial\theta_4)$ and $|G(\vec{\theta})\rangle$ is the ground state of $\mathcal{H}(\vec{\theta})$. The spin-Berry phase is defined as

$$\gamma_i = \frac{1}{i} \int_{L_i} d\vec{\theta} \cdot \vec{A}(\vec{\theta}). \tag{S4}$$

The integration path L_i is given as follows. Defining five points in the parameter space as

$$E_1 = (2\pi, 0, 0, 0),$$
$$E_2 = (0, 2\pi, 0, 0),$$
$$E_3 = (0, 0, 2\pi, 0),$$
$$E_4 = (0, 0, 0, 2\pi),$$
$$G = \frac{1}{4}(2\pi, 2\pi, 2\pi, 2\pi),$$

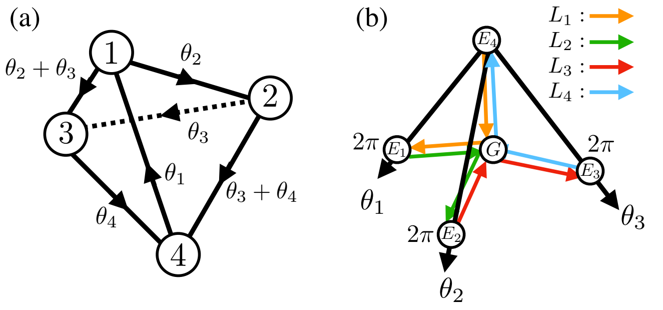

FIG. S1. (a) Peierls phase in a downward tetrahedron of the pyrochlore lattice. (b) Integration paths $L_1, \cdots, L_4$. G represents the center of gravity.

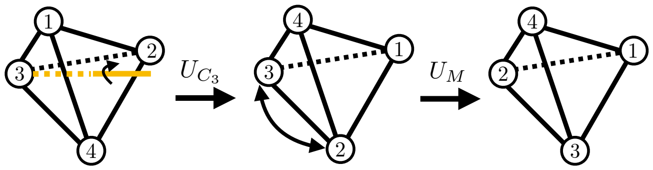

FIG. S2. Unitary operators U_{C_3} and U_M.

we introduce paths from E_i to G as shown in Fig. S1(b) by setting $\phi_4 = 2\pi$, i.e., $\theta_4 = 2\pi - \theta_1 - \theta_2 - \theta_3$. They are expressed as [S3]

$$\vec{f_1}(t) = \frac{2\pi}{4}(4 - 3t, t, t, t),$$
$$\vec{f_2}(t) = \frac{2\pi}{4}(t, 4 - 3t, t, t),$$
$$\vec{f_3}(t) = \frac{2\pi}{4}(t, t, 4 - 3t, t),$$
$$\vec{f_4}(t) = \frac{2\pi}{4}(t, t, t, 4 - 3t),$$

where $0 \le t \le 1$. Along these lines, the integral path is defined as $L_i : E_{i-1} \to G \to E_i$, where $E_0 \equiv E_4$.

Due to the equivalence of the paths $L_1, \cdots, L_4$, the spin-Berry phase is quantized into $\mathbb{Z}_4$. Let us now derive

it in detail. Tetrahedron is invariant for any exchange of the vertexes by the symmetric group S_4 [S1]. This implies that the original Hamiltonian $\mathcal{H}(\vec{\theta}=\vec{0})$ have C_3 symmetry and mirror symmetry with respect to the chosen downward tetrahedron, see Fig. S2. With finite $\vec{\theta}$, the symmetry is broken but instead we have

$$(U_M U_{C_3}) H(\vec{\theta}) (U_M U_{C_3})^{-1} = H(g\vec{\theta}), \tag{S5}$$

where g is the 4×4 unitary matrix satisfying $g\vec{\theta} = (\theta_4, \theta_1, \theta_2, \theta_3)$. Clearly, we have

$$g\vec{f}_i(t) = \vec{f}_{i+1}(t), \tag{S6}$$

where $i = 1, \cdots, 4$ and $\vec{f}_5 \equiv \vec{f}_1$. This implies $\gamma_1 \equiv \gamma_2 \equiv \gamma_3 \equiv \gamma_4 \equiv \gamma \mod 2\pi$ as follows:

$$\begin{aligned}
\gamma_i &= \sum_j \frac{1}{i}\int_{L_i} d\theta_j \langle G(\vec{\theta})|\frac{\partial}{\partial\theta_j}|G(\vec{\theta})\rangle \\
&= \sum_j \frac{1}{i}\int_{L_{i-1}} d\left(\sum_k g_{jk}\theta'_k\right) \times \\
&\qquad \langle G(g\vec{\theta}')|\sum_l (g^{-1})_{lj}\frac{\partial}{\partial\theta'_l}|G(g\vec{\theta}')\rangle \\
&= \sum_{jkl} g_{jk}(g^{-1})_{lj}\frac{1}{i}\int_{L_{i-1}} d\theta'_k \langle G(g\vec{\theta}')|\frac{\partial}{\partial\theta'_l}|G(g\vec{\theta}')\rangle \\
&= \sum_l \frac{1}{i}\int_{L_{i-1}} d\theta'_l \langle G(g\vec{\theta}')|\frac{\partial}{\partial\theta'_l}|G(g\vec{\theta}')\rangle \\
&= \sum_l \frac{1}{i}\int_{L_{i-1}} d\theta'_l \langle G(\vec{\theta}')|\frac{\partial}{\partial\theta'_l}|G(\vec{\theta}')\rangle \\
&= \gamma_{i-1},
\end{aligned}$$

where $\vec{\theta} = g\vec{\theta}'$, and we use $\partial/(\partial\theta'_l)|G(g\vec{\theta}')\rangle = (U_M U_{C_3})^{-1}\partial/(\partial\theta'_l)|G(\vec{\theta}')\rangle$. Since the sum of the loop $L_1, \cdots, L_4$ is equal to zero, implying

$$\sum_i \gamma_i \equiv 0 \mod 2\pi, \tag{S7}$$

we have

$$\gamma \equiv \frac{n}{4}2\pi \mod 2\pi, \tag{S8}$$

where $n = 0, 1, 2, 3$.

Since the quantized value does not change unless the energy gap closes, γ is an adiabatic invariant for gapped topological phases. For $U = 0$, we have $\gamma = \pi$ for the HOTI while $\gamma = 0$ for the band insulator. Let us now demonstrate it based on the decoupled limit. The HOTI phase includes the decoupled system with $t_\triangle = 0$, whose Hamiltonian is given by $\mathcal{H}(\vec{\theta}) = U_-(\vec{\theta})\mathcal{H}_t^{\bigtriangledown}U_-^\dagger(\vec{\theta})$. The spin-Berry connection $\vec{A} = (A_1, \cdots, A_4)$ is calculated as

$$\begin{aligned}
A_j(\vec{\theta}) = \langle G(\vec{\theta})|\frac{\partial}{\partial\theta_j}|G(\vec{\theta})\rangle &= \langle G_0|U_-^\dagger(\vec{\theta})\frac{\partial}{\partial\theta_j}U_-(\vec{\theta})|G_0\rangle \\
&= \langle G_0|\left(i\sum_{k=j}^4 n_k^-\right)|G_0\rangle,
\end{aligned}$$

where $|G_0\rangle = |G(\vec{\theta}=\vec{0})\rangle$. Because of symmetry, we have $\langle G_0|n_1^-|G_0\rangle = \cdots = \langle G_0|n_4^-|G_0\rangle \equiv s$ and

$$A_j(\vec{\theta}) = i(5-j)s. \tag{S9}$$

Consequently, the $\mathbb{Z}_4$ spin-Berry phase is given by

$$\begin{aligned}
\gamma_1 &= \frac{1}{i}\int_{L_1} d\vec{\theta}\cdot\vec{A}(\vec{\theta}) \\
&= \frac{1}{i}\int_0^{2\pi} d\theta_1 A_1(\vec{\theta}) + \frac{1}{i}\int_{2\pi}^0 d\theta_4 A_4(\vec{\theta}) \\
&= \frac{1}{i}\int_0^{2\pi} d\theta_1\,(i4s) + \frac{1}{i}\int_{2\pi}^0 d\theta_4\,(is) \\
&= 6\pi s.
\end{aligned} \tag{S10}$$

As mentioned in the main text, we have $s = -1/2$ in the half-filling, which implies $\gamma = \pi \mod 2\pi$. The other limit, i.e., $t_\bigtriangledown = 0$ is included in the band insulating phase. Its Hamiltonian is given by $H(\vec{\theta}) = H^\triangle$. Because of the independence of $\vec{\theta}$, we have $\gamma = 0 \mod 2\pi$.

S2. COMPUTATIONAL DETAILS OF QMC SIMULATIONS

In the scheme of the auxiliary field QMC, the Suzuki-Trotter decomposition [S4, S5] is first applied to $e^{-\beta\mathcal{H}}$ as $e^{-\beta\mathcal{H}} \simeq \prod e^{-\Delta\tau\mathcal{H}_t/2}e^{-\Delta\tau\mathcal{H}_U}e^{-\Delta\tau\mathcal{H}_t/2}$, where $\mathcal{H}_t$ stands for the noninteracting parts in $\mathcal{H}$, and $\Delta\tau = \beta/M$ is a Trotter slice with M being integer. The discrete Hubbard-Stratonovich transformation [S6] is then applied to each term of $e^{-\Delta\tau U n_{i\uparrow}n_{i\downarrow}}$, introducing an auxiliary Ising-type variable at each spatial site in each imaginary time slice. The summation over the auxiliary fields involving the MN_s Ising variables is performed by Monte Carlo (MC) sampling.

We set the Trotter slice as $\Delta\tau = 0.1$, for which the Trotter errors of order $O(\Delta\tau^2)$ are sufficiently small compared with statistical errors of the MC sampling. As for the Hubbard-Stratonovich transformation, we employ one which couples to the spin degree of freedom. Typically, we perform 4×10^3 MC sweeps for equilibration, followed by 8×10^4 MC sweeps for measurement, which are divided into 20 bins to estimate the statistical error by the standard deviation. Each MC sweep consists of MN_s local updates and N_s global moves [S7]. The simulations are carried out on the finite size clusters with L up to 5 (8) corresponding to $N_\mathrm{s} = 500$ (480) under the PBC (OBC).

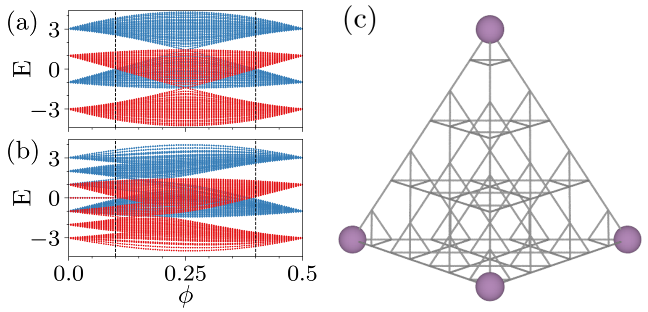

FIG. S3. Energy spectra as function of ϕ at $U = 0$ for (a) $L = 10$ under the PBC and (b) $L = 8$ under the OBC. Red (blue) symbols represent the energy levels for up (down) spin. Vertical dashed lines indicate $\phi^0_{\rm c1} \simeq 0.1$ and $\phi^0_{\rm c2} \simeq 0.4$. (c) The averaged probability densities of the degenerated zero-energy states for $L = 4$ under the OBC are shown by the radius of the purple spheres for $\phi = 0.04$.

S3. ENERGY SPECTRA AT $U = 0$

The energy spectra as function of ϕ in the noninteracting limit are shown in Fig. S3. For the system under the PBC, the single-particle gap opens for the HOTI of $\phi < \phi^0_{\rm c1} \simeq 0.1$ and the BI of $\phi > \phi^0_{\rm c2} \simeq 0.4$. For the system under the OBC, the eightfold degenerate zero-energy states appear only in the HOTI. The averaged probability densities of these degenerated states are shown in Fig. S3(c) for $\phi = 0.04$.

S4. DETERMINATION OF PHASE BOUNDARIES

As shown in Fig. S4, we find $\phi^U_{\rm c1} = 0.15(1)$ and $0.13(1)$ for $U = 3$ and 1. Above $\phi^U_{\rm c1}$, the strong finite-size effect is observed at low T [see Figs. S4(c) and S4(f)], implying the absence of the bulk spin gap.

S5. COLLAPSE OF SPIN GAP

We confirm that the spin gap indeed vanishes at $\phi = \phi^U_{\rm c1}$ from magnetization plateaus under the magnetic field h as shown in Fig. S5. It is noted that the simulations for $h \neq 0$ are possible without encountering the negative sign problem, since the model can be mapped onto the attractive model. The critical magnetic field, $h_{\rm c}$, is determined, in the similar way to $\phi^U_{\rm c1}$, as the point above which $\langle S^z_{\rm tot}\rangle/N_{\rm UC}$ deviates from -1. Since the spin gap is proportional to $h_{\rm c}$, the ϕ-dependence of $h_{\rm c}$ in Fig. S5(c) represents how the spin gap decreases. Thus, the critical point $\phi^U_{\rm c1}$ is estimated as the point of ϕ for which $h_{\rm c}$ is zero. The result in Fig. S5(c) shows that $\phi^U_{\rm c1}$ estimated in this way turn out to agree well with those obtained from the T-dependence of $\langle S^z_{\rm tot}\rangle/N_{\rm UC}$ in Fig. S4 within the error bars.

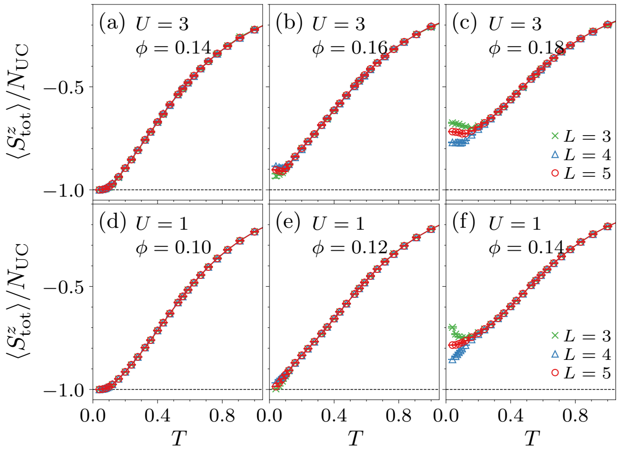

FIG. S4. Temperature dependence of $\langle S^z_{\rm tot}\rangle/N_{\rm UC}$ for various values of ϕ under the PBC. Upper [(a)-(c)] and lower [(d)-(f)] panels show the results of $U = 3$ and $U = 1$, respectively. The horizontal dashed lines indicate $\langle S^z_{\rm tot}\rangle/N_{\rm UC} = -1$, which is the value of the ground-state in the HOTMI phase.

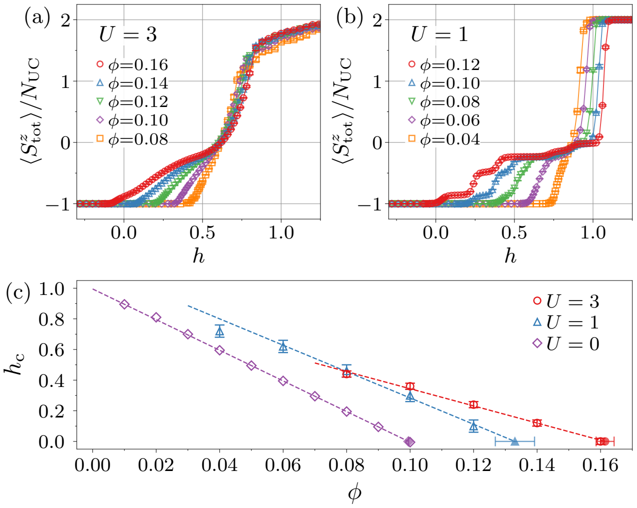

FIG. S5. $\langle S^z_{\rm tot}\rangle/N_{\rm UC}$ as a function of the magnetic field h for (a) $U = 3$ and (b) $U = 1$, and $L = 4$ under the PBC at $T = 0.025$, from which the critical magnetic fields $h_{\rm c}$ are determined. (c) ϕ-dependence of $h_{\rm c}$ for $U = 3$ and 1. For comparison, corresponding exact values for $U = 0$ are also shown. The dashed lines are linear fits to the data points. The filled symbols at $h_{\rm c} = 0$ indicate $\phi^U_{\rm c1}$ estimated from the fittings.

[S1] Y. Hatsugai and I. Maruyama, Euro. Phys. Lett **95**, 200003 (2011).
[S2] K. Kudo, Ph. D. thesis (University of Tsukuba, 2021).
[S3] H. Araki, private communication.

[S4] M. Suzuki, Commun. Math. Phys. **51**, 183 (1976).
[S5] H. F. Trotter, Proc. Am. Math. Soc. **10**, 545 (1959).
[S6] J. E. Hirsch, Phys. Rev. B **28**, 4059 (1983).
[S7] R. Scalettar, R. Noack, and R. Singh, Phys. Rev. B **44**, 10502 (1991).
